# 

**Published:** 1896-04-11

**Authors:** 


### EARLY EDITION.

**Notice to Advertisers and Subscribers.**

All Advertisements, Orders for Copies of The Hospital and Business Communications should be addressed to **THE MANAGER, (Not to The Editor)**, The Hospital, 428, Strand, London, W.C.

The Cost of Subscription, post free, is as follows:—Per week, 2½d.; per quarter, 2s. 8d.; per half-year, 5s. 3d.; per annum, 10s. 6d. Foreign:—Per Annum, 15s. 2d.; payable, in all cases, in advance.

Ready Shortly.

**"Burdett's Hospitals and Charities, 1896."**

"THE HOSPITAL": Editorial and Publishing Offices, 428, Strand, London, W.C.

### TERMS OF SUBSCRIPTION.

### WEEKLY ISSUE.

| | Great Britain. | Foreign and Colonial. |
|---|---|---|
| For One Year ... | 10s. 6d. | 15s. 2d. |
| For Six Months ... | 5s. 3d. | 7s. 7d. |
| For Three Months ... | 2s. 8d. | 3s. 10d. |

### MONTHLY PARTS.

| | Post Free to any part of the World. |
|---|---|
| For One Year ... | 8s. 6d. |
| For Six Months ... | 4s. 3d. |
| For Three Months ... | 2s. 2d. |

Postal Orders and Cheques should be made payable to "The Scientific Press, Limited."

N.B.—In order to prevent delay, all business communications should be addressed to "The Manager" only, and not to any person individually.

"THE HOSPITAL" can be obtained of all Booksellers and Newsagents in the United Kingdom, and at all the Railway Stalls, of Messrs. W. H. Smith and Son; and also from the following Agents in the Colonies and Abroad:—

Paris—Librairie Galignani, 224, Rue de Rivoli.
Berlin—Messrs. A. Asher and Co.
Leipsig—Mr. F. A. Brockhaus.
New York—The International News Co.
Montreal—The Montreal News Co.
Toronto—The Toronto News Co.
Melbourne, Sydney, Adelaide, and New Zealand—Messrs. Gordon and Gotch.
India—At all the Railway Bookstalls and Agencies of Messrs. A. H. Wheeler and Co.
South Africa—Messrs. Juta, Cape Town.

### THE HOSPITAL.

AN INSTITUTIONAL JOURNAL OF THE MEDICAL SCIENCES AND HOSPITAL ADMINISTRATION.

WEEKLY, 2d. | MONTHLY, 6d.

### SUBSCRIPTION FORM.

To the Publishers of "The Hospital,"

428, Strand, London, W.C.

*Kindly enter my name as a Weekly Subscriber to* "**The Hospital**" *for* { Twelve Months / Six Months / Three Months }

*the price for the same being enclosed, viz.,* { Ten Shillings and Sixpence * / Five Shillings and Threepence / Two Shillings and Eightpence } *including postage.*

WRITE CLEARLY.

*Name*____________________

*Titles and Description*____________________

*Address*____________________

*Date*____________________

* For Foreign rates, and rates for Monthly Parts, see above.

### The Student of Medicine.

### APPOINTMENTS.

W. T. D. Allon, M.B., B.S.I., appointed Assistant Medical Officer of the Workhouse of the Parish of Liverpool. Mr. W. Berry, appointed Medical Officer of Health for the Hanley Urban District. G. F. Cross, M.B., B.S., appointed Medical Officer of Health to the Downham Rural District Council. G. F. P. Clapham, L.R.C.P., L.R.C.S.Edin., appointed Assistant Medical Officer of the Workhouse of the Parish of Liverpool. A. Densham, M.R.C.S., L.R.C.P., L.D.S., appointed Assistant Dental Surgeon to the Dental Hospital of London, Leicester Square. R. C. H. Drabble, L.D.S., appointed Dental Surgeon to the Sheffield Royal Hospital. F. Fawssett, M.B.Lond., B.S., M.R.C.S., appointed Medical Officer for the St. John (Without) District of the Lewes Union. Dr. C. Gilchrist, appointed Medical Officer for the Osbournby District of the Sleaford Union. F. Harrison, M.R.C.S., L.D.S., appointed Dental Surgeon to the Sheffield Royal Hospital. R. J. Hughes, L.R.C.P., M.R.C.S., appointed Medical Officer of the Workhouse of the Birkenhead Union. T. St. G. Mivart, M.D., F.R.C.S., L.R.C.P.Edin., appointed a Medical Inspector to the Local Government Board. F. G. Mordaunt, L.D.S., appointed Dental Surgeon to the Sheffield Royal Hospital. P. Paget, L.R.C.P.Lond., M.R.C.S., appointed Medical Officer for the High Halden District of the Tenterden Union. E. C. Pern, M.R.C.S., L.R.C.P.Lond., appointed Medical Officer of the Workhouse and the Droxford and Soberton District of the Droxford Union. J. L. Pike, L.D.S., appointed Dental Surgeon to the Sheffield Royal Hospital. R. Purnell, M.D.St. And., M.R.C.S.Eng., appointed Medical Officer of Health to the Wells Town Council. B. Roth, F.R.C.S.Eng., appointed Surgeon for the Orthopædic Department of the Royal Alexandra Hospital for Children, Brighton. C. Stokes, L.D.S., appointed Dental Surgeon to the Sheffield Royal Hospital. Thorp, M.R.C.S.Eng., L.S.A., appointed Medical Officer for the Fourth District of the Hexham Union. W. B. Tolputt, L.D.S., appointed Dental Surgeon to the Sheffield Royal Hospital. H. T. M. Whitling, M.B., B.S.Durh., M.R.C.S., appointed Medical Officer tor the Third District of the Market Harborough Union.

### VACANCIES.

The following vacancies are announced this week, the amount of salary in each case being given after the name of the institution:—

**Resident Medical Officers.**

Bethlem Hospital.—Two Resident Clinical Assistants. Appointments for six months. Apartments, board, and washing provided. Applications, endorsed "Clinical Assistantship," to the Treasurer, Bethlem Hospital, London, S.E., before April 13th.

City of Birmingham.—Deputy Medical Superintendent for the City Hospital, Little Bromwich, under 40 years of age, unmarried. and doubly qualified. Salary £175 per annum, with residence, rations, and attendance. Applications, endorsed "Deputy Medical Superintendent," to be sent to Mr. J. Keyte, Clerk to the Health Committee, Council House, Birmingham, by April 14th.

Dr. Steevens's Hospital, Dublin.—House Surgeon. Appointment for two years. Salary, £100 per annum, with apartments, fire, and light. Applications to the Governors and Guardians of Dr. Steevens's Hospital by April 11th.

East London Hospital for Children and Dispensary for Women, Glamis Road, Shadwell.—Resident Medical Officer, doubly qualified. Salary, £80 per annum, with board and residence. Applications to Thomas Hayes, Secretary by April 11th.

Hereford County and City Asylum.—Medical Superintendent. Salary £400 per annum, with furnished house, coals, gas, vegetables, and washing. Applications to the Chairman, Asylum Committee, Shirehall, Hereford, by April 28th.

Kesteven and Grantham District Asylum.—Medical Superintendent. Salary £300 a year, with rations, coal, light. and washing. Must devote his whole time to the duties of the office. Applications to Jos. Phillips, Clerk to the Visitors, Stamford, by April 11th.

Liverpool Northern Hospital. Assistant House Surgeon, doubly qualified. Salary £70 per annum, with residence and maintenance in the house. Applications to the Chairman by April 17th.

Manchester Royal Eye Hospital.—House Surgeon. Salary £70 per annum, with residence, board, and washing. Applications, "House Surgeon," to the Chairman of the Board of Management by April 11th.

### THE STUDENT OF MEDICINE—*continued.*

Donegal District Lunatic Asylum, Letterkenny.—Assistant Medical Officer; qualified in medicine, surgery, and midwifery; unmarried, and not more than 30 years old. Salary £100 per annum, with furnished apartments, rations, washing, fuel, light and attendance, valued at £100 per annum. Applications to Dr. Moore, Resident Medical Superintendent, by May 9th.

Miller Hospital and Royal Kent Dispensary, Greenwich Road, S.E.—Junior Resident Medical Officer. Appointment for six months, with prospect of re-election. Salary £30 per annum, with board, attendance, and washing. Applications to the Honorary Secretary or Secretary by April 11th.

Royal Hospital for Diseases of the Chest, City Road, E.C.—Resident Medical Officer. Appointment for six months, but eligible for re-election. Salary at the rate of £100 per annum, with furnished apartments, board, and washing. Applications to the Secretary at the Hospital by April 13th.

**Non-Resident Medical Officers.**

Belgrave Hospital for Children, 77, Gloucester Street, S.W.—Dispenser (male or female). Salary £60 per annum. Also a Clinical Assistant. Applications to the Honorary Secretary by April 11th.

Dental Hospital of London and London School of Dental Surgery, Leicester Square.—Lecturer on Mechanical Dentistry. Applications to Morton Smale, Dean, by May 11th.

National Dental Hospital, Great Portland Street, W.—Anæsthetist. Applications to Ed. Almack, Secretary, by April 15th.

Owens College, Manchester.—Junior Demonstrator in Anatomy. Applications to the Registrar by April 27th.

Royal Orthopædic and Spinal Hospital, Birmingham.—Honorary Assistant Surgeon. Applications to F. J. Abbott, Honorary Secretary, 9, Bennett's Hill, Birmingham, by April 18th.

St. Mary's Hospital Medical School, Paddington.—Bacteriologist. Applications to the Dean by April 11th.

### EAST LONDON HOSPITAL FOR CHILDREN AND DISPENSARY FOR WOMEN, GLAMIS ROAD, SHADWELL, E.

RESIDENT MEDICAL OFFICER.

